# A Biologically Inspired Movement Recognition System with Spiking Neural Networks for Ambient Assisted Living Applications

**DOI:** 10.3390/biomimetics9050296

**Published:** 2024-05-15

**Authors:** Athanasios Passias, Karolos-Alexandros Tsakalos, Ioannis Kansizoglou, Archontissa Maria Kanavaki, Athanasios Gkrekidis, Dimitrios Menychtas, Nikolaos Aggelousis, Maria Michalopoulou, Antonios Gasteratos, Georgios Ch. Sirakoulis

**Affiliations:** 1 Department of Electrical and Computer Engineering, Democritus University of Thrace, 67100 Xanthi, Greece; apassias@ee.duth.gr (A.P.); ktsakalo@ee.duth.gr (K.-A.T.); 2 Department of Production and Management Engineering, Democritus University of Thrace, 67100 Xanthi, Greece; ikansizo@pme.duth.gr (I.K.); agaster@pme.duth.gr (A.G.); 3 School of Physical Education and Sport Science, Democritus University of Thrace, 69100 Komotini, Greece; mkanavak@affil.duth.gr (A.M.K.); agkrekid@phyed.duth.gr (A.G.); dmenycht@affil.duth.gr (D.M.); nagelous@phyed.duth.gr (N.A.); michal@phyed.duth.gr (M.M.)

**Keywords:** ambient assisted living (AAL), spiking neural networks (SNNs), reconfigurable neuromorphic processors, elderly activity recognition, energy-efficient processing, real-time processing, activity monitoring

## Abstract

This study presents a novel solution for ambient assisted living (AAL) applications that utilizes spiking neural networks (SNNs) and reconfigurable neuromorphic processors. As demographic shifts result in an increased need for eldercare, due to a large elderly population that favors independence, there is a pressing need for efficient solutions. Traditional deep neural networks (DNNs) are typically energy-intensive and computationally demanding. In contrast, this study turns to SNNs, which are more energy-efficient and mimic biological neural processes, offering a viable alternative to DNNs. We propose asynchronous cellular automaton-based neurons (ACANs), which stand out for their hardware-efficient design and ability to reproduce complex neural behaviors. By utilizing the remote supervised method (ReSuMe), this study improves spike train learning efficiency in SNNs. We apply this to movement recognition in an elderly population, using motion capture data. Our results highlight a high classification accuracy of 83.4%, demonstrating the approach’s efficacy in precise movement activity classification. This method’s significant advantage lies in its potential for real-time, energy-efficient processing in AAL environments. Our findings not only demonstrate SNNs’ superiority over conventional DNNs in computational efficiency but also pave the way for practical neuromorphic computing applications in eldercare.

## 1. Introduction

Contemporary research conducted by the World Health Organization predicts a significant increase in the elderly population and longer lifespans globally in the coming decades. As more seniors choose to “age in place” instead of moving to nursing homes, eldercare becomes increasingly crucial to supporting their independence and maintaining their health [1,2]. This situation puts significant pressure on the healthcare sector, necessitating the implementation and enhancement of ambient assisted living (AAL) systems [3,4,5,6]. AAL systems monitor movements and detect falls in addition to recognizing activities, gestures, and emotions, and they aim to provide an integrated and effective solution for assisted living.

Current approaches predominantly utilize wearable devices, such as wristbands for motion capture and action recognition; audio devices for recording low-level sounds during daily activities; and marker-based systems, such as Vicon and Qualisys, for accurate 3D pose representation and action recognition [7,8,9]. The emergence of deep neural networks (DNNs) has significantly improved activity recognition in ambient assisted living (AAL) applications [10]. However, these methods are typically characterized by high energy consumption and computational demands, making them less suitable for continuous, real-time applications in domestic environments. In contrast to biological neurons that communicate with spikes, DNNs utilize mathematical calculations between neurons [11,12]. The need for an efficient, adaptive, and less resource-intensive technology is evident, especially one that can accurately recognize and interpret the complex array of human movements that are characteristic of the elderly.

In response to and to address the limitations of DNNs, there is a growing research focus on neuromorphic computing, particularly spiking neural networks (SNNs), which can be implemented in neuromorphic processors and promise a more naturalistic computational paradigm that provides a more energy-efficient approach than DNNs implemented using GPUs [13,14,15]. Despite their potential and advantages, such as fast inference, analog computation, and low energy consumption, the deployment of SNNs in practical AAL applications remains nascent, with several challenges in their non-differentiable nature, learning efficiency, adaptability, and hardware implementation still requiring further investigation before transferring knowledge from classic AAL approaches to SNNs. This study situates itself within this context, aiming to bridge the gap between the potential of neuromorphic computing and its real-world application in eldercare.

To meet the increasing demand for real-time and large-scale neuromorphic processors, previous studies have proposed a reconfigurable neuromorphic model based on field-programmable gate array (FPGA) technology and asynchronous cellular automata [16,17,18,19,20,21,22,23]. These models offer hardware-efficient solutions for various applications, including Parkinson’s treatment emulation, central pattern generation for hexapod robots, spike-timing-dependent synaptic plasticity, neural integrators, tumor immunotherapy, and ergodic cellular automaton neuron models [16,17,18,19]. Implementing these models in FPGAs offers lower power consumption and hardware requirements compared with conventional models. The asynchronous cellular automaton neuron (ACAN) model, initially introduced in [24] and further optimized in [22], reproduces neuromorphic behaviors of cortical neurons using discrete-state dynamics, and it requires fewer hardware resources. Its dynamic adjustability after implementation makes it a versatile and suitable solution for implementing SNNs, and it is ideal for real-time neuromorphic applications [25,26], including movement classification tasks.

The contributions of this paper are multifold. Firstly, we propose using the ACAN model as a building block for neuromorphic networks, validating its ability to reproduce a total of 20 cortical spiking patterns on an FPGA and demonstrating its versatility and robustness in diverse neuromorphic modeling scenarios. Secondly, a comprehensive parametric analysis is demonstrated for the MNIST hand-written digit dataset to identify optimal learning configurations for these neuron models by adjusting both the neuron characteristics and learning parameters, significantly enhancing their learning efficiency and applicability. Thirdly, we apply our methodology to our novel movement dataset and the critical task of human movement classification for recognizing basic distinct elderly human movements, such as gait, cutting, standing up, sitting down, and turning. The adoption of the ACAN model and the ReSuMe learning method has been pivotal in achieving precise, adaptive learning for these specific tasks. Our work has yielded promising results for its integration into real-time, holistic AAL systems.

## 2. Dataset

**Figure 1:**
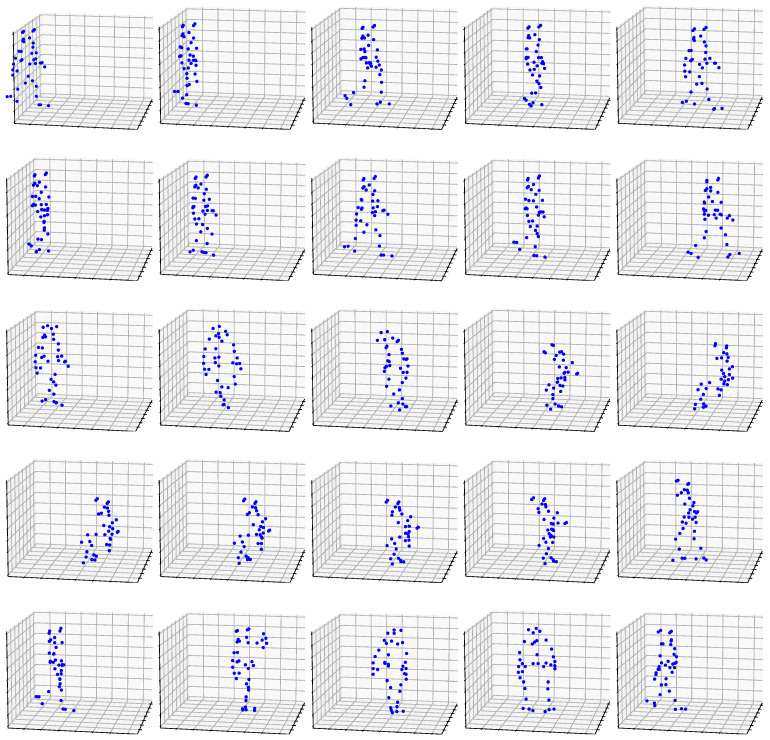
Evolving from left to right, the five action scenarios are illustrated in each row, i.e., (1) *cutting*, (2) *gait*, (3) *sitting down*, (4) *standing up*, and (5) *turning*.

In this section, we describe the data collection methods, dataset division, and preprocessing steps used to train the models discussed in the next section, namely, Section 3. The study involved two senior individuals who repeated five basic action scenarios: *cutting*, *gait*, *sitting down*, *standing up*, and *turning* (Figure 1). The Vicon system assessed the individuals’ posture by tracking the position of $n_{j}$ joints in the human body, which were represented by 3D coordinates (*x*, *y*, *z*) and sampled at a consistent rate of 10 ms. The data underwent post-processing and were saved as *.c3d* files, containing joint coordinates, metadata, sensor setups, and specific measurements. After collecting action scenarios for all individuals, we chose to analyze $n_{j}=38$ principal joints. This decision was made since other, additional joints often had missing estimations caused by occlusions during particular activities.

Mokka software, which is a tool for analyzing motion kinematics and kinetics [27], was used to transform the *.c3d* files into a tabular format and remove any unnecessary metadata. To maintain consistency, markers that displayed inconsistency or were absent were removed. In order to handle composite movement labels, we conducted manual annotation of the crucial frames that signified a shift in the type of movement, leading to distinct "areas" of movement within the same sample. Given the dataset’s constrained and imbalanced nature, the instances were partitioned into segments of fixed length by using a sliding window technique. Segments that intersected with annotated areas were categorized based on their corresponding movement, while the remaining segments were classified as *gait*. The dataset was expanded from 126 to 17,803 samples. In order to maintain balanced classes and prevent any class imbalance, we restricted the number of samples per class to match the size of the smallest classes for experimentation in Section 4. The dataset was further enhanced by calculating velocity and acceleration data. The samples were then normalized to a range of [0,1] as spiking rates. These rates will be provided in the subsequent section.

The basic movement types of *cutting*, *gait*, *sitting down*, *standing up*, *and turning* encompass a combination of gait and labeled movement as described by Menychtas [28]. The original samples were evaluated manually to identify pivotal frames that indicated the shift between various sorts of movements, resulting in the aforementioned 17,803 data points. The samples were divided into segments by using a sliding window of size *S* frames, and the frames were categorized into their respective classes. This class was determined either as *gait* or as the specific movement indicated by the label of the original data point. The determination was based on whether the window included the critical area, as identified by the critical frames. More precisely, samples labeled *cutting* and *turning* exhibited a solitary critical area in the middle of the movement. On the other hand, samples labeled *standing up* and *sitting down* featured critical areas at the onset of the movement. Notably, the samples classified as gait did not display any critical areas. After the preprocessing step, the number of samples increased, as indicated in Table 1.

## 3. Method

This section provides an overview of the approach, which encompasses the ACAN model, the *ReSuMe* training method, and the network configuration.

### 3.1. Asynchronous Cellular Automaton-Based Neuron

The asynchronous cellular automaton-based neuron (ACAN) model is a digital neuron designed for field-programmable gate array (FPGA) optimization, having the ability to replicate various neural activities.

**Figure 2:**
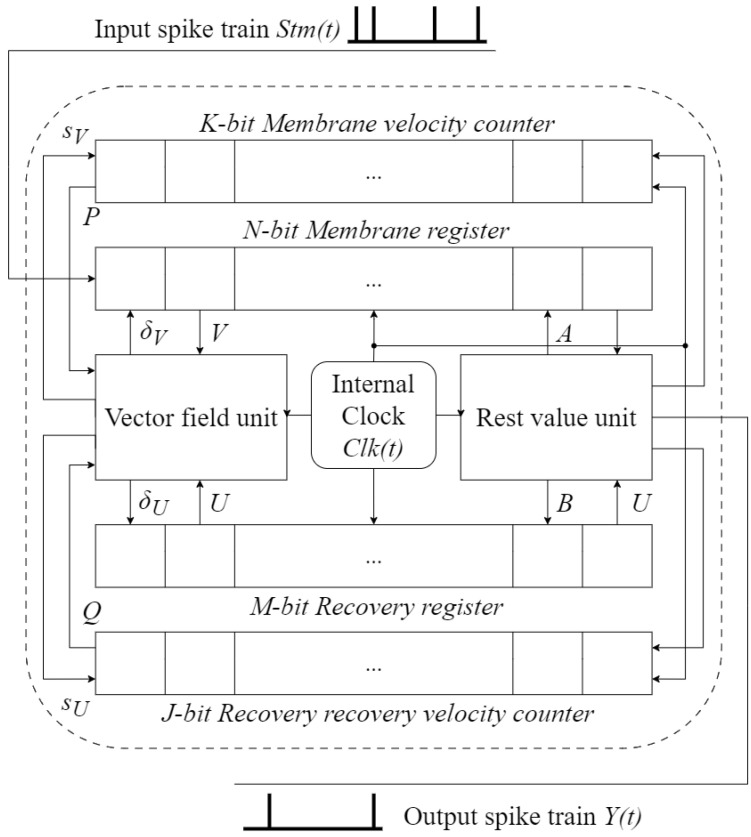
Generalized asynchronous cellular automaton-based neuron (ACAN) model adapted from [22].

The ACAN architecture, proposed by Matsubara and Torikai in their publication [24], is a digital neuron model that draws inspiration from the Izhikevich model. It is specifically designed for digital systems, with a particular emphasis on its compatibility with FPGAs. The model replicates a range of spiking and bursting patterns observed in cortical neurons [29,30]. The ACAN operates in a generalized configuration where it receives action potentials (spikes) as input, represented by Stm(t), modifies its internal variables, and produces output spikes (Y(t)). Every ACAN unit is equipped with an internal clock (Clk), enabling asynchronous operations among ACAN units as they function independently of a global clock signal (Figure 2).

The internal state of an ACAN unit is represented by the following four bidirectional shift registers with positive integer bit lengths of *N*, *M*, *K*, and *J*, respectively:

- The membrane register is an *N*-bit bidirectional shift register with an integer state *V* in the range of (0,…,N−1), representing the membrane potential of the neuron model.
- The recovery register is an *M*-bit bidirectional shift register with an internal state *U* in the range of (0,…,M−1), representing the recovery variable of the neuron model.
- The membrane velocity counter is a *K*-bit register with an internal state *P* in the range of (0,…,K−1), controlling the velocity of membrane potential *V*.
- The recovery velocity counter is a *J*-bit register with an internal state *Q* in the range of (0,…,J−1), controlling the velocity of recovery variable *U*.

Furthermore, the ACAN’s expected behavior is determined by two logic units, namely, the Vector Field Unit and the Rest Value Unit, which do not retain any memory.

- The Vector Field Unit determines the vector field characteristics for states *V* and *U*.
- The Rest Value Unit sets the rest values for states *V* and *U*.

Each field unit comprises logic gates and reconfigurable wires that provide connections between the membrane and rest registers.

The control signals $(s_{V},s_{U})\in 0,1$ and $\left(\delta_{V},\delta_{U}\right)\in \left\{-1,0,1\right\}$, which are generated by the Vector Field Unit, are defined as follows:

(1) $$\begin{array}{c} s_{V}=\left\{\begin{array}{cc} 1 & \mathrm{if}\;P\geq P_{h}\left(V,U\right)\\ 0 & \mathrm{otherwise} \end{array}\right.s_{U}=\left\{\begin{array}{cc} 1 & \mathrm{if}\;Q\geq Q_{h}\left(V,U\right)\\ 0 & \mathrm{otherwise} \end{array}\right. \end{array}$$

(2) $$\delta_{V}=D_{V}\left(V,U\right),\delta_{U}=D_{U}\left(V,U\right)$$

(3) $$F(V,U)=N(\gamma_{1}{\left(V/N-\gamma_{2}\right)}^{2}+\gamma_{3}-U/M)/\lambda ,$$

(4) $$G(V,U)=\mu M(\gamma_{4}\left(V/N-\gamma_{2}\right)+\left(\gamma_{3}+\gamma_{5}\right)-U/M)\lambda ,$$

(5) $$P_{h}\left(V,U\right)=\left\lfloor |F^{-1}\left(V,U\right)|\right\rfloor -1,$$

(6) $$Q_{h}\left(V,U\right)=\left\lfloor |G^{-1}\left(V,U\right)|\right\rfloor -1,$$

(7) $$D_{V}\left(V,U\right)=sgn(F(V,U)),$$

(8) $$D_{U}\left(V,U\right)=sgn(G(V,U))$$

The rest value unit generates two signals (A,B) that determine the reset values of the (V,U) states after a reset is triggered.

(9) $$A=\lfloor \rho_{1}N\rfloor ,$$

(10) $$B(U)=U+\lfloor \rho_{2}M\rfloor$$

where $\left(\rho_{1},\rho_{2}\right)$ are parameters.

The ACAN’s dynamics are characterized by nine hyper-parameters, namely, $M,N,K,J,\Gamma =(\gamma_{1},\gamma_{2},\gamma_{3},\gamma_{4},\gamma_{5},\lambda ,\mu ,\rho_{1},\rho_{2})$ [22,24]. The aforementioned parameters are pivotal for the variety of spiking patterns that are achievable by the ACAN. These patterns are described in detail in Table 2 and are documented in the study by Matsubara et al. [31]. The ACAN configuration types are commonly utilized in the ensuing parametric analysis.

In a nutshell, the ACAN model functions in the following manner: Input is received by the ACAN in the form of binary spikes, which are weighted by synaptic weight. After considering the input and its previous state, the unit carries out calculations based on Equations (1)–(10), altering its internal state and generating a new output. In this study, we focus on monitoring the basic parameter *V*, which represents the membrane potential of a physical neuron.

### 3.2. Remote Supervised Method (ReSuMe)

The remote supervised method (ReSuMe) [32], which was used for SNN training, is described along with its adaptation for digital settings. The ReSuMe is a learning method that relies on the synaptic plasticity rule introduced by Hebb in his publication [33]. In order to successfully modify ReSuMe for a digital environment, we aimed to closely replicate the procedure in a specifically digital format. As a result, it was necessary to deviate from the continuous-time formulation described in [34] and modify it to suit digital implementation. We compute the difference between the values of $S^{d}\left(t\right)$ and $S^{l}\left(t\right)$ by using discrete time intervals. This involves transforming the output (post-synaptic) and desired spike trains, $S^{l}\left(t\right)$ and $S^{d}\left(t\right)$, into their discrete time-series counterparts, $S^{l}\left[n\right]$ and $S^{d}\left[n\right]$. At each time step *n*, there is either a spike (1) or no spike (0) in this format. The output at time $n_{i}$ is determined based on the occurrence of spikes in $S^{l}\left[n_{i}\right]$ and $S^{d}\left[n_{i}\right]$. If a spike occurs in $S^{l}\left[n_{i}\right]$ but not in $S^{d}\left[n_{i}\right]$, the output is 1. If a spike occurs in $S^{d}\left[n_{i}\right]$ but not in $S^{l}\left[n_{i}\right]$, the output is −1. If spikes occur in both $S^{l}\left[n_{i}\right]$ and $S^{d}\left[n_{i}\right]$, the output is 0. This digital adaption enables concurrent processing across the full frame, making it more suitable for our system’s real-time operation, where inputs are received as distinct binary spikes rather than spike timing.

In particular, ReSuMe modifies the synaptic weight (*w*) between a pre-synaptic neuron ($n^{in}$) and a post-synaptic neuron ($n^{l}$) based on a target spike train, a pre-synaptic spike train, and a post-synaptic spike train, i.e., $S^{d}\left(t\right),S^{in}\left(t\right)$, and $S^{l}\left(t\right)$, respectively, with the following rule:

(11) $$\frac{d}{dt}w\left(t\right)=\left[S^{d}\left(t\right)-S^{l}\left(t\right)\right]\left[a+\int_{0}^{\infty }W\left(s\right)S^{in}\left(t-s\right)ds\right]$$

The aforementioned equation computes the alteration in each synaptic weight (*w*). The learning rate (lr) controls the ultimate adjustment of weights. In this instance, the variable *a* denotes the magnitude of the non-correlation component’s influence on the total weight alteration. The following convolution represents the alterations of *w* through the Hebbian-like process. The learning window, denoted by the integral kernel W(s), represents the convolution’s kernel. It is defined based on the time delay (*s*) between the spiking events occurring among neurons. The form of the learning window (W(s)) bears resemblance to the one described in spike-timing-dependent plasticity (STDP) [34]. In our implementation of the ReSuMe algorithm, we computed the exponential window, *W*, in advance in order to optimize the speed of our learning process, thus achieving faster processing times. The convolution in the equation above, $LW=e^{-\Delta t/\tau }$, was computed for each spike in the input spike trains ($S^{in}\left(t\right)$). Here, Δt represents the time difference between a prior spike and the current time (*t*) in the spike train.

The final form of the learning rule employed is the following one:

(12) $$w^{\prime }=w+lr\left(S^{d}-S^{l}\right)\cdot \left(\alpha +LW\right)$$

where *w* is the synaptic weight matrix of each connection, lr is the learning rate, $S^{d}$ and $S^{l}$ are the desired and the post-synaptic spike trains matrices for all available neurons, α is the non-correlation amplitude term, and LW is the pre-calculated learning window. Equation (12) achieves parallelization by performing vector–matrix multiplication between the output matrix, $\left(S^{d}-S^{l}\right)$, and the pre-calculated learning window matrix, LW, both of which have the same dimension representing time step *S*. This subtle and sophisticated technique enhances the effectiveness and adaptability of learning, enabling seamless integration of the ReSuMe method into digital neuromorphic systems.

### 3.3. Network Architecture

**Figure 3:**
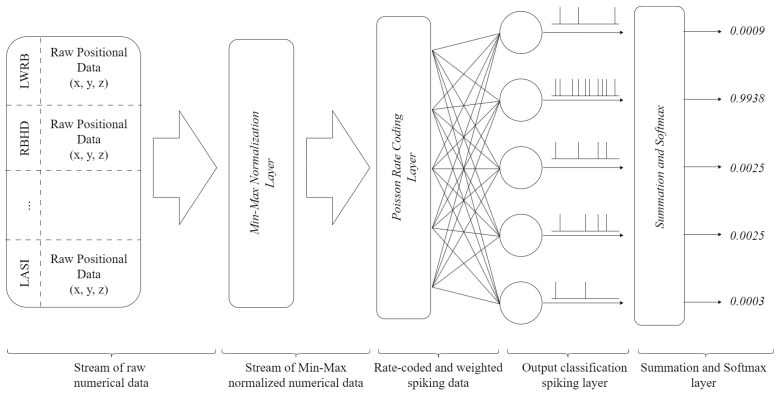
ACAN network architecture.

The architecture of the SNN, encompassing its layers and the procedure of converting data into spikes, is elucidated. The proposed SNN consists of three main layers, as illustrated in Figure 3: an input layer, where data are fed into the network; a rate-coding layer, where data are transformed into a format suitable for neurons (i.e., spikes); and a classification layer composed of ACANs with uniform registers of size *R*, which also functions as the output layer. This layer conducts a comparison between the real outputs and the predicted outputs of the neurons. During both training and testing, performance is evaluated by using a summation and softmax layer, which is implemented as described in [35,36]. The data are displayed as time intervals of positional x, y, and z data for thirty-eight (38) distinct markers positioned on the individuals’ bodies. This particular dataset is unsuitable for utilization with SNNs, hence necessitating its conversion into spike-based representations.

To accomplish this, we employ the inhomogeneous Poisson process for spike generation [37]. This procedure utilizes normalized data within the range of [0,1] as time-dependent spike rates. Every feature within the frame is allocated a distinct random number from a uniform distribution, which is subsequently compared to that particular feature. A spike is recorded at the specific time if the drawn number does not exceed the characteristic. The spike trains are next fed into five (5) ACANs, which categorize them based on one of five movement classifications (Figure 1). After each training sample, the network’s training accuracy is assessed by comparing its pre-training output to the ground truth. This is feasible because the network utilizes the complete output for a specific input sample during training, guaranteeing that the data are essentially unknown, particularly during the first epoch.

After the training process, a test phase is conducted when the network identifies the appropriate label by considering the neuron with the highest spiking rate, which is the most active one. In the last layer, the output of every neuron is added up over the whole sample time period. This is then processed through a softmax layer to find the output with the most spikes, which is then assumed to be the network’s output. Subsequently, each instance is juxtaposed with the ground truth, and every accurate prediction increases a counter. Following each training period, the network’s overall accuracy is computed in the following manner:

accuracy=correct/totalsamples

The suggested architecture offers a comprehensive framework for efficiently converting and processing data by using the proposed SNN, enabling precise movement classification in active and assisted living (AAL) applications, as explained in the following section.

## 4. Experiments and Results

### 4.1. ACAN Spiking Activity Reproduction on an FPGA

**Figure 4:**
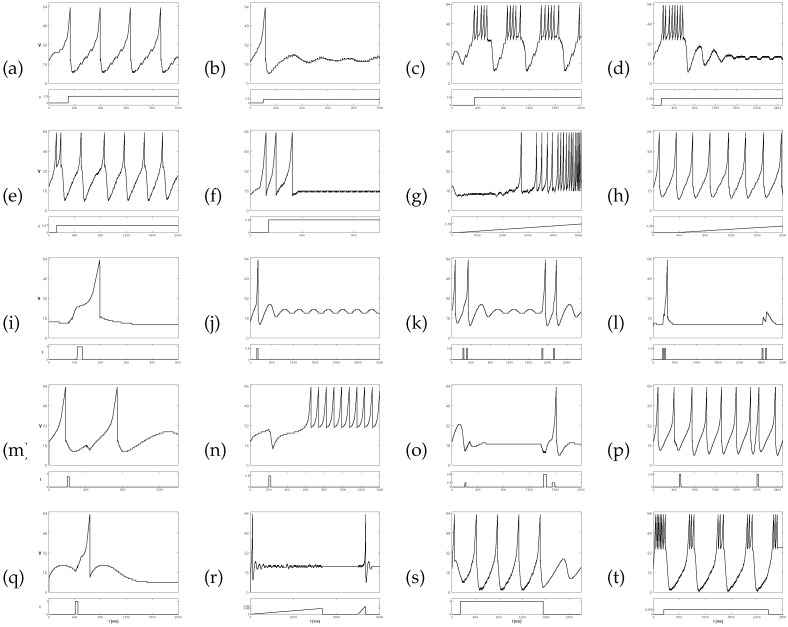
The various spiking patterns reproduced by using our VHDL-implemented ACAN model in the ModelSim environment. The patterns are organized from left to right and from top to bottom: (**a**) tonic spiking, (**b**) phasic spiking, (**c**) tonic bursting, (**d**) phasic bursting, (**e**) mixed-mode spiking, (**f**) spike frequency adaptation, (**g**) class 1 excitation, (**h**) class 2 excitation, (**i**) spike latency, (**j**) sub-threshold oscillation, (**k**) resonator, (**l**) integrator, (**m**) rebound spike, (**n**) rebound burst, (**o**) threshold variability, (**p**) bistability, (**q**) depolarizing after-potential, (**r**) accommodation, (**s**) inhibition-induced spiking, and (**t**) inhibition-induced bursting.

We assessed the feasibility of the ACAN model by implementing it and replicating its cortical spiking processes by using the ModelSim environment. The VHDL-coded ACAN model was developed to empirically showcase its capacity to accurately replicate a range of neuronal responses, as depicted in Figure 4. The model’s adaptability was demonstrated by reproducing various patterns of spiking and bursting activities, which were based on the ACAN types listed in Table 2. These spiking and bursting activities are as follows: (a) tonic spiking, (b) phasic spiking, (c) tonic bursting, (d) phasic bursting, (e) mixed-mode spiking, (f) spike frequency adaptation, (g) class 1 excitation, (h) class 2 excitation, (i) spike latency, (j) sub-threshold oscillation, (k) resonator, (l) integrator, (m) rebound spike, (n) rebound burst, (o) threshold variability, (p) bistability, (q) depolarizing after-potential, (r) accommodation, (s) inhibition-induced spiking, and (t) inhibition-induced bursting.

### 4.2. MNIST Hand-Written Digit Dataset

In order to evaluate the capabilities of the SNN, we performed an initial experiment by utilizing the MNIST hand-written digit dataset [38]. Our ACAN-based SNN architecture’s fundamental learning capabilities were initially validated by using the MNIST dataset as a proof of concept. Next, we concentrated on a new collection of movement data acquired by using the Vicon motion capture device. This dataset showcases the system’s capability to transition from recognizing patterns in images to analyzing intricate time-series data that represent human movements. We utilized MATLAB to train the network by using a dataset consisting of 60,000 training photos and 10,000 test images. The 28-pixel-by-28-pixel input images were transformed into spike trains of 30 time steps apiece, with the pixel values determining the spiking rates. This resulted in 784×30 samples. The ACAN parameters that were adjusted for maximum accuracy are as follows: Vector Γ assumed the values (7,0.3,0.2,2.8,0.06,1,0.7,0.3,0), and the register size was R=64 for all registers. The network was trained by using ReSuMe, with a learning rate of 0.0001 and τ of 15. Following training, the network was tested by using new images and achieved an accuracy of 89%, which is comparable to prior research in the field of SNNs [26], demonstrating the network’s capacity to understand spatio-temporal correlations within the data.

### 4.3. Network and Training Optimization

**Figure 5:**
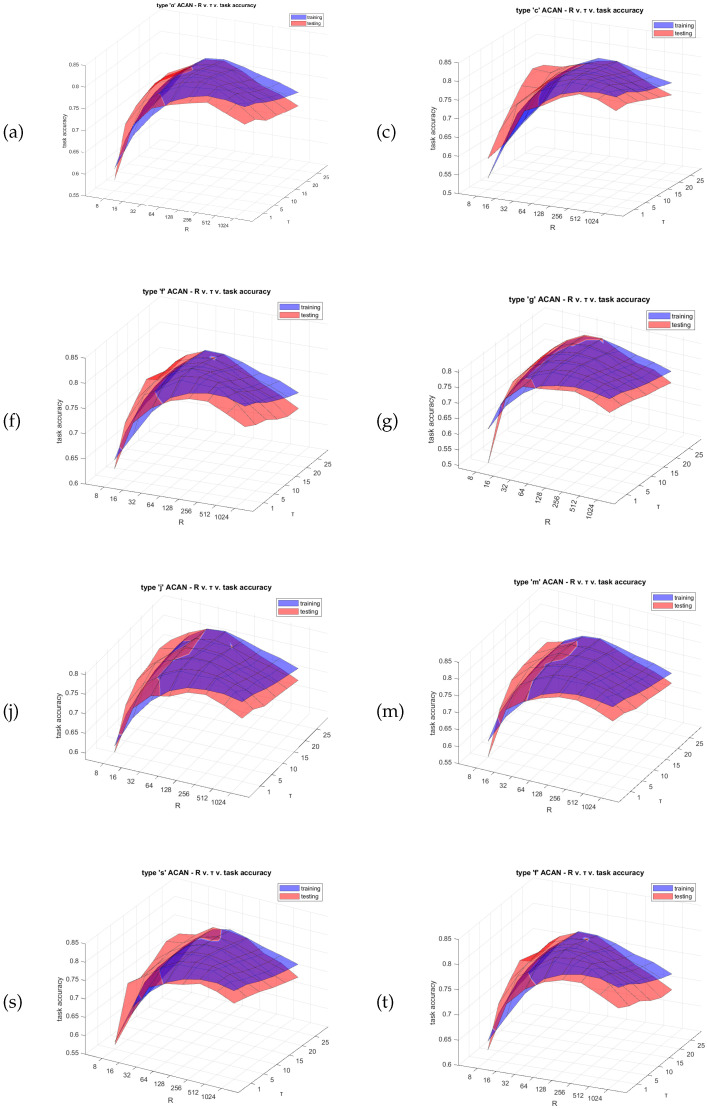
Parametric analysis of different ACAN neuron types for the MNIST hand-written digit dataset. The analysis was carried out by adjusting the register range (*R*), time constant (τ), and ACAN neuron configuration parameters. Left to right, top to bottom: (**a**,**c**,**f**,**g**,**j**,**m**,**s**,**t**) ACAN model parameters were used as presented in Table 2. The figures illustrate the influence of varying the size of the ACAN’s register (*R*) and the time constant (τ) of ReSuMe on the training accuracy (in **blue**) and the test accuracy (in **red**).

An in-depth parametric analysis was carried out to fine-tune the network’s hyperparameters, including the register size (*R*) of the ACAN model neuron, the time constant of ReSuMe, and the overall type of the ACAN model (please refer to Table 2), and to evaluate the impact of spiking activity patterns on learning effectiveness. The analysis was performed in MATLAB, evaluating several ACAN models by systematically varying each parameter, such as register sizes and time constants, within a specified range. The type of ACAN includes the set of the following parameters: {a,c,f,g,j,m,s,t}. For the size of the register (*R*), we tested the values {8,16,32,64,128,256,512,1024}. Finally, for the time constant (τ), we tested the values {1,5,10,15,20,25}. The data shown in Figure 5 and Table 3 indicate that the ACAN parameters play a crucial role in determining the system’s learning capability. Figure 5 illustrates the substantial impact of various ACAN parameters on the learning process of the system. This refers to how the neuron’s different spiking patterns affect the learning process. The sub-figures of Figure 5 are distinctively unique from each other, especially in terms of their visual characteristics. Table 3 displays the optimal performance achieved in both training and testing by the parametric analysis. The ideal setup consisted of the *t* type ACAN with a register size (*R*) of 128 and a time constant (τ) of 10. This setup achieved an accuracy of 80.12% on the test set. The data shown in Table 3 help us choose the optimal settings for our system considering the limitations of the particular application we are dealing with.

### 4.4. Novel Movement Dataset

**Figure 6:**
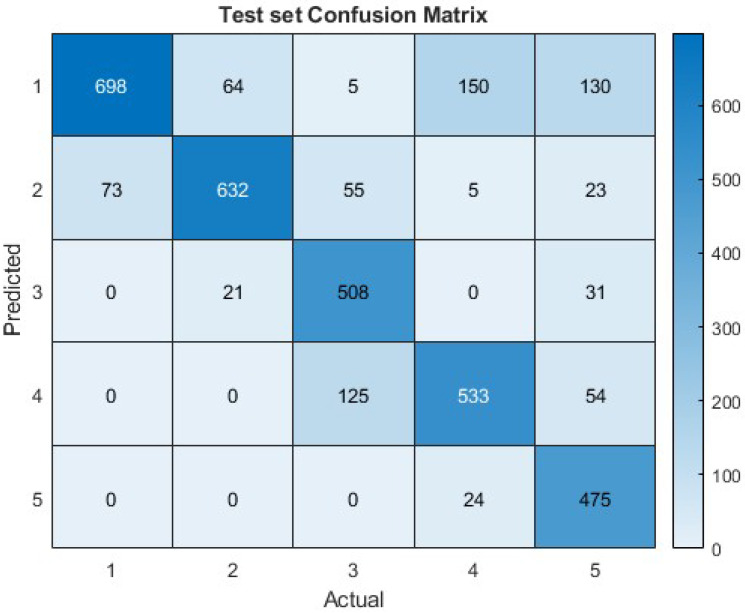
Confusion matrix of SNN performance on the test set of the first experiment with a prediction accuracy of 78.9%.

We applied the ACAN spiking network to the new movement dataset, following the preprocessing steps described in Section 2. The samples were set to be S=100 frames long, resulting in the creation of matrices sized 114×100. The normalized data were converted into spike trains and utilized to train the SNN by using a specified 90/10 training/test split. Incorporating velocity data enhanced performance and led to input matrices of dimensions 228×100. The MNIST experiment utilized certain settings for training by using ReSuMe: τ=15, α=1, and lr=0.0001, respectively. The training lasted for 75 epochs. The SNN accurately identified new data points with an accuracy of 78.9%, as shown in Figure 6.

The accuracy scores achieved were satisfactory, as demonstrated in comparable research cited in [39]; nonetheless, several factors might impact performance. The number of data is a crucial factor, since a larger dataset can lead to a more effective classifier. The network’s single-layer architecture may restrict its performance; multi-layered spiking neural networks often achieve higher accuracy. The model’s ability to learn may be restricted by the training method. Sequential data in a real-world scenario are likely to be highly correlated due to sharing most frames and belonging to the same sample class, perhaps resulting in more accurate results. The specific moment when a significant movement starts and the moments around it are not dependable indicators of the type of movement occurring might be crucial to enhancing classification accuracy.

**Figure 7:**
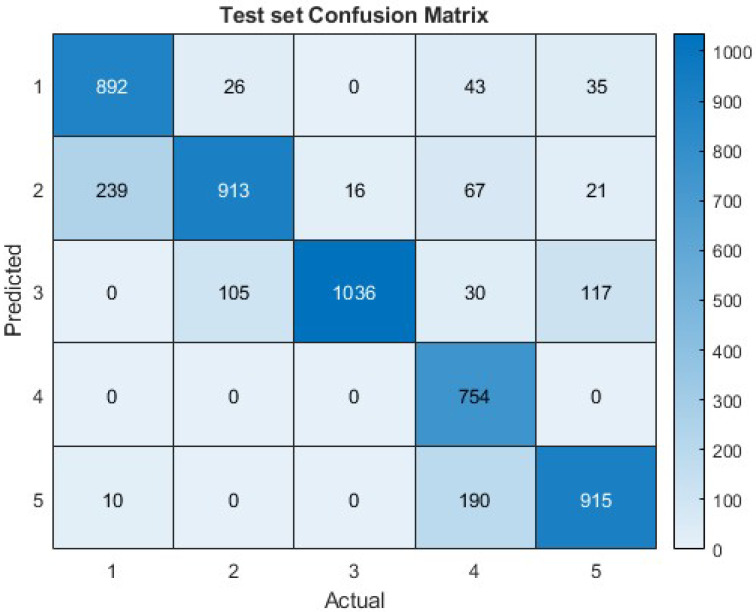
Confusion matrix of SNN performance on the test set of the second experiment with a prediction accuracy of 83.3%.

Another approach to enhancing the network’s performance was attempted by considering input features as distinct and autonomous, similar to how the image’s pixels were handled in the MNIST hand-written digit classification experiment. With the identical conditions as the MNIST experiment, but with 10 time step units per input instead of 30, the input size increased to an 11,400 × 10 matrix, which is a thousand times larger than that in the previous experiment. This has a substantial impact on execution times (about ∼2800 seconds each epoch). By using an 85/15 training/test split, the data setup achieved a test accuracy of 83.3%, indicating a 4.5% improvement, as shown in Figure 7.

## 5. Conclusions and Future Work

The study has successfully demonstrated the effectiveness of the ACAN model within the domain of SNNs for AAL applications, emphasizing its precision and versatility in elderly activity recognition. This research focuses on experimentally validating the ACAN model’s capability to reproduce several types of known neural spiking activity, showcasing its promise for diverse neuromorphic applications. The study incorporates a thorough parametric analysis to pinpoint essential settings for enhancing learning speed and accuracy in various circumstances. The experiment we conducted on human movement categorization by using SNNs showcased the model’s robust capabilities and offered valuable insights into sample processing and training techniques. The experiment obtained an accuracy of 83.3% in classifying five unique types of motions. This accuracy is regarded satisfactory considering the limitations of FPGA-based implementation and the novelty of the dataset. It is also in line with findings from comparable research as found in the literature [39]. Moreover, the results show the promise of neuromorphic computing in the field of AAL.

Our method shows great promise for real-time applications in fields that require low-energy, real-time processing, such as wearable technology, edge computing, and robotics. Although the reported accuracy limits are notable, they offer valuable guidance for enhancing future model versions. This study significantly contributes to the area of SNNs and human-based movement classification, establishing a foundation for future advancements in real-time, energy-efficient computing systems.

In the future, we aim to improve the ACAN model’s performance and relevance. Future research will focus on integrating continuous learning algorithms to tackle the discrete aspects of the training process and enhance logical continuity between subsequent samples. In addition, attempts will be made to expand the model’s relevance to a broader spectrum of motions and situations, encompassing complex, unstructured environments seen in actual AAL settings. Investigating the compatibility with various neuromorphic hardware and sensors, along with assessing the possibilities for scaling and personalization to meet particular user requirements, will be essential. Our goal is to close the gap between the present constraints and the extensive capabilities of SNNs in real-time, adaptive, and energy-efficient applications.

## Figures and Tables

**Table 1:** Number of samples by type of movement before and after preprocessing. After preprocessing the original samples, their quantity increased significantly, allowing for more robust training.

	Before Preprocessing	After Preprocessing
Gait	32	3659
Cutting	50	2404
Standing up	22	2751
Sitting down	20	3286
Turning	12	5703
Total	126	17,803

**Table 2:** ACAN types based on spiking activity and corresponding parameter values.

Type	$\gamma_{1}$	$\gamma_{2}$	$\gamma_{3}$	$\gamma_{4}$	$\gamma_{5}$	λ	μ	$\rho_{1}$	$\rho_{2}$
**a**	7	0.3	0.2	2.8	0.06	*R*	0.7	0.3	0
b	7	0.3	0.2	2.8	0.06	*R*	0.7	0.3	0
**c**	7	0.3	0.2	2.8	0.06	*R*	0.7	0.55	−0.2
d	7	0.3	0.2	2.8	0.06	*R*	0.7	0.55	−0.2
e	7	0.3	0.2	2.8	0.06	*R*	0.7	0.55	−0.2
**f**	7	0.3	0.2	1.1	0.03	*R*	0.01	0.2	0.15
**g**	7	0.3	0.2	−0.5	0.05	*R*	4	0.25	0.4
h	7	0.3	0.2	3	−0.09	*R*	0.5	0.3	0
i	7	0.3	0.2	−0.5	0.05	*R*	4	0.25	0.4
**j**	7	0.3	0.2	3	−0.09	*R*	0.5	0.3	0
k	7	0.3	0.2	3	−0.09	*R*	0.5	0.3	0
l	7	0.3	0.2	−0.5	0.05	*R*	4	0.25	0.4
**m**	7	0.3	0.2	3	−0.1	*R*	0.5	0.3	0
n	7	0.3	0.2	3	−0.1	*R*	0.5	0.48	−0.42
o	7	0.3	0.2	3	0.1	*R*	0.5	0.3	0
p	7	0.3	0.2	3	−0.11	*R*	0.5	0.3	0
q	7	0.3	0.2	−0.5	0.15	*R*	0.5	0.2	−0.3
r	7	0.3	0.2	2.8	0.06	*R*	0.7	0.3	0
**s**	7	0.3	0.5	−5	0	*R*	−0.1	0.4	0.3
**t**	7	0.3	0.5	−5	0	*R*	−0.1	0.55	−0.1

**Table 3:** Accuracy results for various ACAN neuron types on the novel movement dataset. Each type has an accuracy (%) score, as well as values for the register size (*R*) and time constant (τ).

ACAN Model	Training			Testing		
Types	R	τ	Accuracy %	R	τ	Accuracy %
a	128	5	80.75	128	10	79.7
c	256	5	80.87	128	10	79.62
f	128	5	80.77	64	10	79.53
g	128	5	80.87	128	10	79.53
j	128	5	80.87	128	10	79.86
m	128	5	80.81	256	5	79.53
s	256	5	80.79	256,128	5, 10	79.87
**t**	256	5	80.86	**128**	**10**	**80.12**

## Data Availability

The datasets presented in this article are not readily available because the data are part of an ongoing study. Requests to access the datasets should be directed to G.C.S.

## Abbreviations

The following abbreviations are used in this manuscript:

AAL	ambient assisted living
SNN	spiking neural network
DNN	deep neural network
ACAN	asynchronous cellular automata-based neuron
ReSuMe	remote supervised learning
RC	Reservoir Computing
MCG	Magnetocardiogram
EEG	Electroencephalogram
FPGA	field-programmable gate array
STDP	spike-timing-dependent plasticity
VHDL	VHSIC Hardware Description Language
VHSIC	Very-High-Speed Integrated Circuit
MNIST	Modified National Institutes of Standards and Technology
NN	neural network
